# Supplementary material for: Thermal expansion of FeWO4 (ferberite) and FeWO4:Fe2WO6 (7:1): a comparative X-ray and neutron diffraction study
Source: J Appl Crystallogr. 2026 Jul 24;59(Pt 4):1238–46. doi: 10.1107/S1600576726005297 (PMC13440050; doi:10.1107/S1600576726005297)
Supplement: Supplementary file 1 [file j-59-01238-sup1.pdf]

JOURNAL OF
APPLIED
CRYSTALLOGRAPHY

Volume 59 (2026)

Supporting information for article:

Thermal expansion of FeWO4 (ferberite) and FeWO4:Fe2WO6 (7:1): a comparative X-ray and neutron diffraction study

O. Fabelo, L. Cañadillas-Delgado, D. Vie, E. Matesanz, J. Gonzalez-Platas and D. Errandonea

Ferberite crystal structure

Figure S1: Schematic representation of Ferberite structure. Tungsten (iron) atoms and shown in grey (brown) and oxygen atoms in red. The WO6 and FeO6 octahedra are represented.

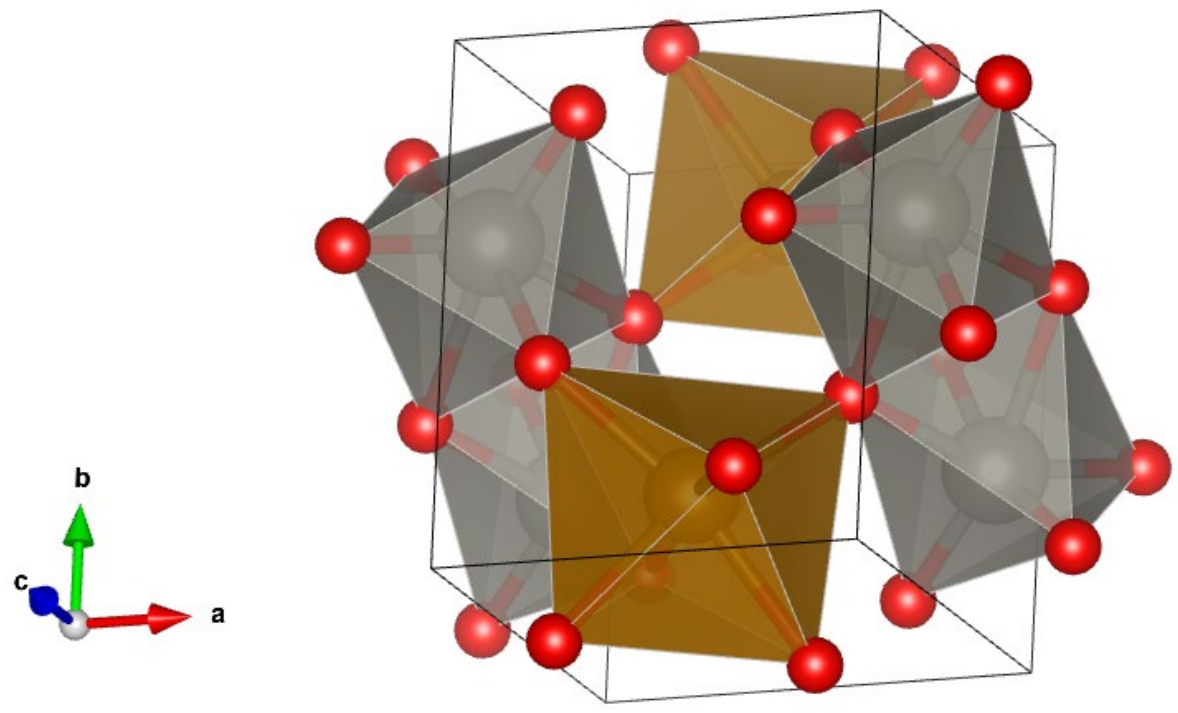

Ferberite - Single Crystal Diffraction

Table S1. Crystallographic data and structure refinement for Ferberite

Empirical formula	$FeWO_4$	$FeWO_4$
Formula weight	303.685	303.685
T/K	**40.0(5)**	**60.0(5)**
λ/Å	0.56087	0.56087
Crystal system	Monoclinic	Monoclinic
Space group	*P2/c*	*P2/c*
a /Å	4.7358(3)	4.7352(3)
b /Å	5.7092(3)	5.7100(3)
c /Å	4.9562(3)	4.9573(3)
α /°	90	90
β /°	90.156(4)	90.154(3)
γ /°	90	90
Volume /$Å^3$	134.003(14)	134.035(14)
Z	2	2
ρ_{calcd}/g cm^{-3}	7.526	7.525
μ/mm^{-1}	25.803	25.797
F(000)	264.3	264.3
Crystal size /mm^3	0.10 x 0.07 x 0.05	0.10 x 0.07 x 0.05
2θ range for data collection/°	5.64 to 68.24	5.64 to 68.28
Index ranges	$-6 \leq h \leq 9$<br>$-11 \leq k \leq 11$<br>$-9 \leq l \leq 9$	$-6 \leq h \leq 9$<br>$-11 \leq k \leq 11$<br>$-9 \leq l \leq 9$
Reflect. collected	5012	5235
Independent reflect. [*R*(int)]	1119 [0.0805]	1116 [0.0773]
Data/restraints/ parameters	1119/0/30	1116/0/30
Goodness-of-fit on F^2	1.078	0.956
Final *R* indices [$I > 2\sigma(I)$]	R_1 = 0.0819, wR_2 = 0.2086	R_1 = 0.0709, wR_2 = 0.1818
R indices (all data)	R_1 = 0.0840, wR_2 = 0.2144	R_1 = 0.0725, wR_2 = 0.1853
Largest diff. peak and hole/e $Å^{-3}$	1.16 /-1.30	1.21 /-1.41

Table S1 (continuation). Crystallographic data and structure refinement for Ferberite

Empirical formula	$FeWO_4$	$FeWO_4$
Formula weight	303.685	303.685
T/K	**80.0(5)**	**100.0(5)**
λ/Å	0.56087	0.56087
Crystal system	Monoclinic	Monoclinic
Space group	*P2/c*	*P2/c*
a /Å	4.7338(5)	4.7361(6)
b /Å	5.7111(6)	5.7078(7)
c /Å	4.9577(6)	4.9590(6)
α /°	90	90
β /°	90.147(4)	90.094(6)
γ /°	90	90
Volume /$Å^3$	134.03(14)	134.06(3)
Z	2	2
ρ_{calcd}/g cm^{-3}	7.525	7.523
μ/mm^{-1}	25.797	25.793
F(000)	264.3	264.3
Crystal size /mm^3	0.10 x 0.07 x 0.05	0.10 x 0.07 x 0.05
2θ range for data collection/°	5.62 to 68.26	5.64 to 68.26
Index ranges	$-6 \leq h \leq 9$<br>$-11 \leq k \leq 11$<br>$-9 \leq l \leq 9$	$-6 \leq h \leq 9$<br>$-11 \leq k \leq 11$<br>$-9 \leq l \leq 9$
Reflect. collected	5412	5545
Independent reflect. [*R*(int)]	1119 [0.0766]	1118 [0.0742]
Data/restraints/ parameters	1119/0/30	1118/0/30
Goodness-of-fit on F^2	0.963	0.907
Final *R* indices [$I > 2\sigma(I)$]	R_1 = 0.0567, wR_2 = 0.1522	R_1 = 0.0540, wR_2 = 0.1423
R indices (all data)	R_1 = 0.0585, wR_2 = 0.1550	R_1 = 0.0567, wR_2 = 0.1536
Largest diff. peak and hole/e $Å^{-3}$	0.86 /-0.75	0.73 /-0.90

Table S1 (continuation). Crystallographic data and structure refinement for Ferberite

Empirical formula	$FeWO_4$	$FeWO_4$
Formula weight	303.685	303.685
T/K	**130.0(5)**	**160.0(5)**
λ/Å	0.56087	0.56087
Crystal system	Monoclinic	Monoclinic
Space group	*P*2/*c*	*P*2/*c*
a /Å	4.7365(5)	4.7375(5)
b /Å	5.7073(7)	5.7074(6)
c /Å	4.9614(6)	4.9622(5)
α /°	90	90
β /°	90.073(4)	90.057(5)
γ /°	90	90
Volume /$Å^3$	134.12(3)	134.12(3)
Z	2	2
ρ_{calcd}/g cm^{-3}	7.526	7.525
μ/mm^{-1}	25.780	25.780
F(000)	264.3	264.3
Crystal size /mm^3	0.10 x 0.07 x 0.05	0.10 x 0.07 x 0.05
2θ range for data collection/°	5.64 to 68.24	5.64 to 68.24
Index ranges	$-6 \le h \le 9$<br>$-11 \le k \le 11$<br>$-9 \le l \le 9$	$-6 \le h \le 9$<br>$-11 \le k \le 11$<br>$-9 \le l \le 9$
Reflect. collected	5417	5318
Independent reflect. [*R*(int)]	1120 [0.0716]	1122 [0.0730]
Data/restraints/ parameters	1120/0/30	1122/0/30
Goodness-of-fit on F^2	0.959	0.931
Final *R* indices [$I > 2\sigma(I)$]	R_1 = 0.0522, wR_2 = 0.1364	R_1 = 0.0550, wR_2 = 0.1480
R indices (all data)	R_1 = 0.0532, wR_2 = 0.1381	R_1 = 0.0559, wR_2 = 0.1497
Largest diff. peak and hole/e $Å^{-3}$	1.0 /-0.82	0.97 /-0.82

Table S1 (continuation). Crystallographic data and structure refinement for Ferberite

Empirical formula	$FeWO_4$	$FeWO_4$
Formula weight	303.685	303.685
T/K	**200.0(5)**	**250.0(5)**
λ/Å	0.56087	0.56087
Crystal system	Monoclinic	Monoclinic
Space group	*P2/c*	*P2/c*
a /Å	4.7397(6)	4.7394(3)
b /Å	5.7082(7)	5.7125(4)
c /Å	4.9647(6)	4.9686(3)
α /°	90	90
β /°	90.028(7)	90.004(4)
γ /°	90	90
Volume /$Å^3$	134.32(3)	134.519(15)
Z	2	2
ρ_{calcd}/g cm^{-3}	7.509	7.498
μ/mm^{-1}	25742	25.704
F(000)	264.3	264.3
Crystal size /mm^3	0.10 x 0.07 x 0.05	0.10 x 0.07 x 0.05
2θ range for data collection/°	5.64 to 68.20	5.62 to 68.16
Index ranges	$-6 \leq h \leq 9$<br>$-11 \leq k \leq 11$<br>$-9 \leq l \leq 9$	$-6 \leq h \leq 9$<br>$-11 \leq k \leq 11$<br>$-9 \leq l \leq 9$
Reflect. collected	5447	5552
Independent reflect. [*R*(int)]	1126 [0.0788]	1124 [0.0819]
Data/restraints/ parameters	1126/0/30	1124/0/30
Goodness-of-fit on F^2	0.934	0.948
Final *R* indices [$I > 2\sigma(I)$]	R_1 = 0.0614, wR_2 = 0.1562	R_1 = 0.0514, wR_2 = 0.1373
R indices (all data)	R_1 = 0.0626, wR_2 = 0.1586	R_1 = 0.0534, wR_2 = 0.1403
Largest diff. peak and hole/e $Å^{-3}$	0.72 /-1.12	0.86 /-0.83

Table S1 (continuation). Crystallographic data and structure refinement for Ferberite

Empirical formula	$FeWO_4$	$FeWO_4$
Formula weight	303.685	303.685
T/K	**300.0(5)**	**350.0(5)**
λ/Å	0.56087	0.56087
Crystal system	Monoclinic	Monoclinic
Space group	*P2/c*	*P2/c*
a /Å	4.7418(1)	4.7425(2)
b /Å	5.7156(2)	5.7174(2)
c /Å	4.9712(2)	4.9733(2)
α /°	90	90
β /°	89.9660(17)	89.924 (2)
γ /°	90	90
Volume /$Å^3$	134.731(8)	134.850(9)
Z	2	2
ρ_{calcd}/g cm^{-3}	7.486	7.479
μ/mm^{-1}	25.663	25.641
F(000)	264.3	264.3
Crystal size /mm^3	0.10 x 0.07 x 0.05	0.10 x 0.07 x 0.05
2θ range for data collection/°	5.62 to 68.22	5.62 to 68.22
Index ranges	$-6 \leq h \leq 9$<br>$-11 \leq k \leq 11$<br>$-9 \leq l \leq 9$	$-6 \leq h \leq 9$<br>$-11 \leq k \leq 11$<br>$-9 \leq l \leq 9$
Reflect. collected	5621	5664
Independent reflect. [*R*(int)]	1124 [0.0634]	1126 [0.0701]
Data/restraints/ parameters	1124/0/30	1126/0/30
Goodness-of-fit on F^2	0.971	0.972
Final *R* indices [$I > 2\sigma(I)$]	$R_1 = 0.0447$, $wR_2 = 0.1202$	$R_1 = 0.0459$, $wR_2 = 0.1212$
R indices (all data)	$R_1 = 0.0457$, $wR_2 = 0.1215$	$R_1 = 0.0472$, $wR_2 = 0.1225$
Largest diff. peak and hole/e $Å^{-3}$	0.72 /-0.60	0.75 /-0.55

Table S1 (continuation). Crystallographic data and structure refinement for Ferberite

Empirical formula	$FeWO_4$	$FeWO_4$
Formula weight	303.685	303.685
T/K	**400.0(5)**	**450.0(5)**
λ/Å	0.56087	0.56087
Crystal system	Monoclinic	Monoclinic
Space group	*P2/c*	*P2/c*
a /Å	4.7432(2)	4.7461(3)
b /Å	5.7195(3)	5.7245(4)
c /Å	4.9754(2)	4.9784(3)
α /°	90	90
β /°	89.887 (3)	89.838(4)
γ /°	90	90
Volume /$Å^3$	134.976(11)	135.258(15)
Z	2	2
ρ_{calcd}/g cm^{-3}	7.472	7.457
μ/mm^{-1}	25.617	25.563
F(000)	264.3	264.3
Crystal size /mm^3	0.10 x 0.07 x 0.05	0.10 x 0.07 x 0.05
2θ range for data collection/°	5.62 to 68.14	5.62 to 68.24
Index ranges	$-6 \leq h \leq 9$<br>$-11 \leq k \leq 11$<br>$-9 \leq l \leq 9$	$-6 \leq h \leq 9$<br>$-11 \leq k \leq 11$<br>$-9 \leq l \leq 9$
Reflect. collected	5619	5678
Independent reflect. [*R*(int)]	1122 [0.0739]	1127 [0.0751]
Data/restraints/ parameters	1122/0/30	1127/0/30
Goodness-of-fit on F^2	0.974	0.967
Final *R* indices [$I > 2\sigma(I)$]	R_1 = 0.0463, wR_2 = 0.1210	R_1 = 0.0445, wR_2 = 0.1201
R indices (all data)	R_1 = 0.0479, wR_2 = 0.1230	R_1 = 0.0466, wR_2 = 0.1232
Largest diff. peak and hole/e $Å^{-3}$	0.62 /-0.59	0.56 /-0.52

Table S1 (continuation). Crystallographic data and structure refinement for Ferberite

Empirical formula	$FeWO_4$
Formula weight	303.685
T/K	**500.0(5)**
λ/Å	0.56087
Crystal system	Monoclinic
Space group	*P2/c*
a /Å	4.7474(4)
b /Å	5.7266(5)
c /Å	4.9798 (4)
α /°	90
β /°	89.790(5)
γ /°	90
Volume /Å^3	135.38(2)
Z	2
ρ_{calcd}/g cm^{-3}	7.450
μ/mm^{-1}	25.540
F(000)	264.3
Crystal size /mm^3	0.10 x 0.07 x 0.05
2θ range for data collection/°	5.62 to 68.00
Index ranges	$-6 \le h \le 9$<br>$-11 \le k \le 11$<br>$-9 \le l \le 9$
Reflect. collected	5049
Independent reflect. [R(int)]	1107 [0.0745]
Data/restraints/ parameters	1107/0/30
Goodness-of-fit on F^2	0.974
Final R indices [$I > 2\sigma(I)$]	R_1 = 0.0549, wR_2 = 0.1485
R indices (all data)	R_1 = 0.0591, wR_2 = 0.1565
Largest diff. peak and hole/e Å^{-3}	0.82 /-0.83

Ferberite Sample – SC-Xray

Table S2: Distances between W-O and Fe-O vs function of temperature.

T(K)	W-O1	W-O2	W-O1a	Fe-O1	Fe-O2	Fe-O2a
40.0(5)	1.908(7)	1.796(7)	2.126(7)	2.068(7)	2.134(7)	2.169(7)
60.0(5)	1.909(6)	1.795(6)	2.125(6)	2.070(6)	2.135(6)	2.173(6)
80.0(5)	1.914(5)	1.789(6)	2.128(5)	2.061(5)	2.135(5)	2.182(6)
100.0(5)	1.912(5)	1.795(5)	2.132(5)	2.062(5)	2.129(5)	2.177(5)
130.0(5)	1.910(4)	1.795(5)	2.130(5)	2.065(5)	2.134(4)	2.172(5)
160.0(5)	1.913(4)	1.793(5)	2.133(5)	2.061(5)	2.133(5)	2.179(5)
200.0(5)	1.912(5)	1.797(5)	2.128(5)	2.069(5)	2.131(5)	2.178(6)
250.0(5)	1.911(4)	1.789(5)	2.129(5)	2.069(5)	2.138(5)	2.186(5)
300.0(5)	1.916(4)	1.792(5)	2.133(5)	2.065(4)	2.134(4)	2.189(5)
350.0(5)	1.917(4)	1.791(5)	2.133(4)	2.063(5)	2.138(4)	2.190(5)
400.0(5)	1.914(4)	1.793(5)	2.131(5)	2.069(5)	2.141(4)	2.188(5)
450.0(5)	1.920(5)	1.788(5)	2.137(5)	2.064(5)	2.141(5)	2.197(5)
500.0(5)	1.911(5)	1.792(5)	2.134(6)	2.075(6)	2.141(6)	2.198(6)

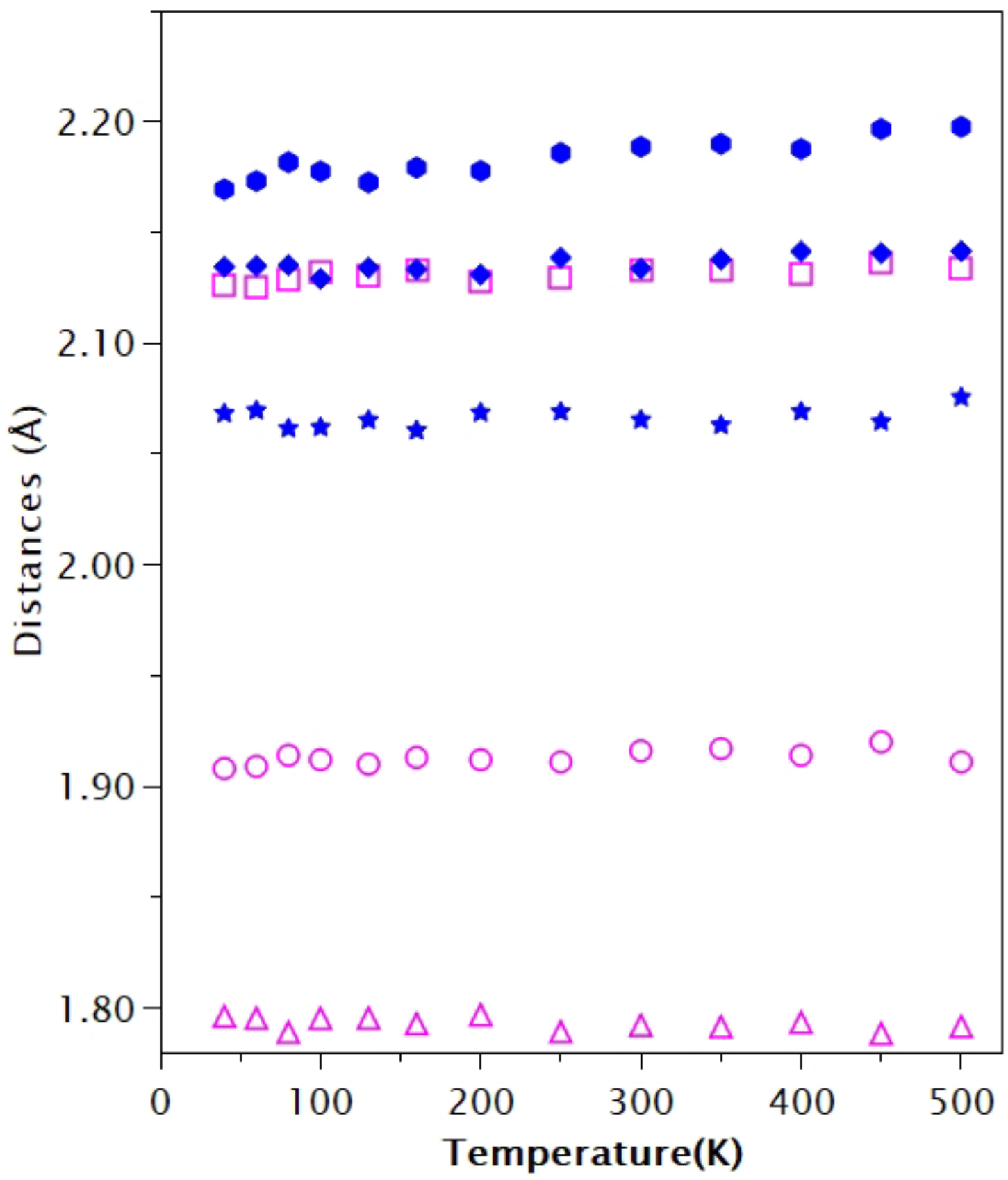

Figure S2: Distances evolution with temperature for Fe-O (blue fill symbols) and W-O (magenta empty symbols). Error bars are smaller than the size symbols.

Ferberite Sample – SC-Xray

Table S3: Unit-cell parameters evolution with temperature.

T(K)	*a(Å)*	*b(Å)*	*c(Å)*	β(º)
40.0(5)	4.7358(3)	5.7092(3)	4.9562(3)	90.156(4)
60.0(5)	4.7352(3)	5.7100(3)	4.9573(3)	90.154(4)
80.0(5)	4.7338(5)	5.7111(6)	4.9577(6)	90.147(4)
100.0(5)	4.7361(6)	5.7078(7)	4.9590(6)	90.094(6)
130.0(5)	4.7365(5)	5.7073(7)	4.9614(5)	90.073(4)
160.0(5)	4.7375(5)	5.7074(6)	4.9622(5)	90.057(5)
200.0(5)	4.7397(6)	5.7082(7)	4.9647(6)	90.028(7)
250.0(5)	4.7394(3)	5.7125(4)	4.9686(3)	90.004(4)
300.0(5)	4.7418(2)	5.7156(2)	4.9712(2)	89.966(2)
350.0(5)	4.7425(2)	5.7174(2)	4.9733(2)	89.924(2)
400.0(5)	4.7432(2)	5.7195(3)	4.9754(2)	89.887(3)
450.0(5)	4.7461(3)	5.7245(4)	4.9784(3)	89.838(4)
500.0(5)	4.7474(4)	5.7266(5)	4.9798(4)	89.790(5)

Ferberite Sample – XRD

Table S4: Unit-cell parameters evolution with temperature from 15-523 K.

T(K)	a*(Å)*	b*(Å)*	c*(Å)*	β(º)
15(1)	4.73425(13)	5.70661(11)	4.94901(14)	90.254(3)
35(1)	4.73413(13)	5.70706(11)	4.94839(14)	90.255(3)
45(1)	4.73377(14)	5.70698(11)	4.94909(14)	90.235(3)
55(1)	4.73432(14)	5.70766(12)	4.94949(14)	90.242(3)
65(1)	4.73395(13)	5.70765(11)	4.94977(14)	90.259(3)
75(1)	4.73371(14)	5.70807(11)	4.95063(14)	90.252(3)
85(1)	4.73412(13)	5.70801(11)	4.95154(14)	90.234(3)
100(1)	4.73421(13)	5.70828(12)	4.95169(14)	90.249(3)
115(1)	4.73451(13)	5.70859(12)	4.95191(14)	90.254(3)
130(1)	4.73508(14)	5.70849(14)	4.95360(15)	90.198(3)
145(1)	4.73492(14)	5.70824(11)	4.95333(15)	90.177(3)
160(1)	4.73558(13)	5.70832(12)	4.95488(15)	90.176(3)
180(1)	4.73676(13)	5.70877(13)	4.95705(16)	90.114(4)
200(1)	4.73688(13)	5.70913(11)	4.95680(15)	90.140(3)
220(1)	4.73826(13)	5.71014(12)	4.96059(15)	90.178(3)
240(1)	4.73864(13)	5.71050(12)	4.96061(15)	90.150(3)
280(1)	4.73975(13)	5.71122(11)	4.96025(15)	90.128(3)
290(1)	4.73975(13)	5.71176(12)	4.96384(15)	90.160(3)
298(1)	4.74053(14)	5.71213(14)	4.96472(15)	90.154(5)
300(1)	4.74053(12)	5.71213(12)	4.96472(15)	90.154(3)
323(1)	4.74262(25)	5.71266(25)	4.96520(29)	90.145(6)
373(1)	4.74313(27)	5.71382(26)	4.96457(27)	90.221(6)
423(1)	4.74569(25)	5.71690(25)	4.96664(24)	90.317(5)
473(1)	4.74769(24)	5.71887(25)	4.96884(23)	90.347(4)
523(1)	4.74912(23)	5.72079(25)	4.97096(26)	90.339(5)

LeBail Refinements

T(K)	R_p	R_{wp}	R_{exp}	χ^2	R_{Bragg}
15-523	14.8-15.7	19.5-21.1	11.88-12.34	2.70-2.99	1.93-2.38

Table S5: Unit-cell parameters evolution with temperature from 298-1123 K.

T(K)	a(Å)	b(Å)	c(Å)	β(º)
298(1)	4.7377(10)	5.7104(11)	4.9650(12)	90.087(4)
323(1)	4.7405(12)	5.7115(12)	4.9649(13)	90.169(3)
373(1)	4.7415(12)	5.7129(12)	4.9666(14)	90.184(3)
423(1)	4.7441(12)	5.7156(12)	4.9694(14)	90.202(3)
473(1)	4.7465(12)	5.7175(12)	4.9720(14)	90.238(2)
523(1)	4.7488(12)	5.7200(12)	4.9751(14)	90.265(2)
573(1)	4.7505(12)	5.7219(12)	4.9760(14)	90.294(18)
623(1)	4.7523(12)	5.7243(12)	4.9785(14)	90.321(18)
673(1)	4.7539(12)	5.7263(12)	4.9801(14)	90.355(17)
723(1)	4.7561(12)	5.7283(12)	4.9824(14)	90.384(17)
773(1)	4.7577(13)	5.7305(12)	4.9839(15)	90.409(16)
823(1)	4.7597(13)	5.7329(12)	4.9858(15)	90.437(16)
873(1)	4.7614(13)	5.7348(12)	4.9870(15)	90.463(16)
923(1)	4.7629(13)	5.7373(12)	4.9885(14)	90.495(15)
973(1)	4.7645(13)	5.7388(12)	4.9906(15)	90.519(15)
1023(1)	4.7656(12)	5.7407(12)	4.9917(15)	90.538(15)
1073(1)	4.7675(12)	5.7432(11)	4.9933(13)	90.554(15)
1123(1)	4.7683(12)	5.7444(12)	4.9944(14)	90.582(15)

LeBail Refinements

T(K)	R_p	R_{wp}	R_{exp}	χ^2	R_{Bragg}
298-1123	13.6-16.1	17.8-21.0	10.56-10.68	2.79-3.92	1.74-2.22

$FeWO_4$ Synthesized Sample – XRD

Table S6: Unit-cell parameters evolution with temperature from 15-523 K.

T(K)	a(Å)	b(Å)	c(Å)	β(º)
15(1)	5.6954(3)	5.6920(3)	4.9408(3)	89.895(7)
25(1)	5.6959(3)	5.6926(3)	4.9414(3)	89.883(7)
35(1)	5.6959(3)	5.6925(3)	4.9412(3)	89.870(7)
45(1)	5.6959(3)	5.6925(3)	4.9423(3)	89.874(7)
55(1)	5.6964(3)	5.6930(3)	4.9423(3)	89.860(6)
65(1)	5.6954(3)	5.6920(3)	4.9415(3)	89.847(6)
75(1)	5.6955(3)	5.6921(4)	4.9424(3)	89.833(6)
85(1)	5.6949(3)	5.6915(4)	4.9419(3)	89.846(6)
100(1)	5.6955(3)	5.6921(4)	4.9424(3)	89.844(6)
115(1)	5.6961(3)	5.6927(4)	4.9431(3)	89.840(6)
130(1)	5.6968(3)	5.69344)	4.9436(3)	89.848(6)
145(1)	5.6966(3)	5.6932(4)	4.9437(3)	89.838(6)
160(1)	5.6968(3)	5.6934(4)	4.9441(3)	89.843(6)
180(1)	5.6963(3)	5.6929(4)	4.9442(3)	89.842(6)
200(1)	5.6974(3)	5.6940(4)	4.9452(3)	89.832(6)
220(1)	5.6976(3)	5.6942(4)	4.9457(3)	89.833(6)
240(1)	5.6984(3)	5.6950(4)	4.9460(3)	89.821(6)
260(1)	5.6987(3)	5.6953(4)	4.9465(3)	89.819(6)
280(1)	5.6986(3)	5.6952(4)	4.9463(4)	89.811(6)
290(1)	5.6994(3)	5.6960(4)	4.9470(4)	89.810(6)
300(1)	5.6997(3)	5.6963(3)	4.9475(3)	89.808(7)
323(1)	5.7015(3)	5.6982(3)	4.9490(3)	89.800(7)
373(1)	5.7032(3)	5.6999(3)	4.9501(3)	89.801(7)
423(1)	5.7048(3)	5.7014(3)	4.9512(3)	89.799(7)
473(1)	5.7063(3)	5.7029(3)	4.9530(3)	89.797(6)
523(1)	5.7069(3)	5.7035(3)	4.9539(3)	89.791(6)

LeBail Refinements

T(K)	R_p	R_{wp}	R_{exp}	χ^2	R_{Bragg}
15-523	10.8-14.7	16.0-21.9	12.13-12.22	1.72-3.24	0.07-0.31

Table S7: Unit-cell parameters evolution with temperature from 298-1123 K.

T(K)	a(Å)	b(Å)	c(Å)	β(º)
298(1)	4.6861(3)	5.6903(3)	4.9408(3)	89.964(8)
323(1)	4.6872(3)	5.6907(3)	4.9418(3)	89.964(7)
373(1)	4.6889(3)	5.6925(3)	4.9433(3)	89.957(7)
423(1)	4.6898(3)	5.6942(3)	4.9444(3)	89.958(7)
473(1)	4.6914(3)	5.6958(3)	4.9455(3)	89.955(7)
523(1)	4.6933(3)	5.6973(3)	4.9473(3)	89.954(6)
573(1)	4.6948(3)	5.6979(3)	4.9482(3)	89.947(6)
623(1)	4.6958(3)	5.6994(3)	4.9494(3)	89.937(6)
673(1)	4.6969(3)	5.7011(3)	4.9506(3)	89.933(6)
723(1)	4.6976(3)	5.7021(4)	4.9521(3)	89.928(7)
773(1)	4.6988(3)	5.7033(4)	4.9531(3)	89.926(7)
823(1)	4.7004(4)	5.7048(4)	4.9551(3)	89.840(7)
873(1)	4.7043(4)	5.7070(4)	4.9584(3)	89.773(6)
923(1)	4.7070(4)	5.7085(4)	4.9598(4)	89.749(6)
973(1)	4.7112(4)	5.7109(4)	4.9629(4)	89.701(6)
1023(1)	4.7148(4)	5.7122(4)	4.9657(4)	89.681(6)
1073(1)	4.7203(4)	5.7156(4)	4.9707(4)	89.629(5)
1123(1)	4.7235(4)	5.7178(5)	4.9725(4)	89.600(5)

LeBail Refinements

T(K)	R_p	R_{wp}	R_{exp}	χ^2	R_{Bragg}
298-1123	11.8-13.4	15.9-18.4	10.44-10.54	2.28-3.12	0.08-0.30

$FeWO_4$ Synthesized Sample – Neutrons

Table S8: Unit-cell parameters evolution with temperature.

T(K)	a(Å)	b(Å)	c(Å)	β(º)
1.97(3)	4.68883(9)	5.69197(14)	4.94270(12)	90.180(3)
9.85(5)	4.68878(9)	5.69189(14)	4.94266(12)	90.179(3)
17.74(5)	4.68881(9)	5.69184(14)	4.94271(12)	90.177(3)
29.69(5)	4.68876(9)	5.69185(14)	4.94288(12)	90.175(3)
39.75(5)	4.68856(9)	5.69199(14)	4.94308(11)	90.172(3)
49.87(5)	4.68854(9)	5.69215(14)	4.94337(11)	90.169(3)
59.88(5)	4.68833(9)	5.69244(14)	4.94377(11)	90.164(3)
69.79(11)	4.68843(9)	5.69254(13)	4.94408(11)	90.159(3)
79.84(6)	4.68841(9)	5.69290(13)	4.94432(11)	90.158(3)
89.66(16)	4.68859(9)	5.69280(14)	4.94450(12)	90.153(3)
99.30(40)	4.68884(9)	5.69296(14)	4.94478(11)	90.154(3)
118.50(90)	4.68901(9)	5.69328(14)	4.94508(14)	90.145(3)
138.1(1.1)	4.68955(9)	5.69337(14)	4.94552(11)	90.143(3)
157.9(1.1)	4.69005(9)	5.69368(14)	4.94608(11)	90.141(3)
177.8(1.2)	4.69060(9)	5.69392(14)	4.94659(11)	90.133(3)
198.3(7)	4.69116(8)	5.69445(13)	4.94743(11)	90.127(3)
217.8(1.2)	4.69172(8)	5.69464(13)	4.94787(11)	90.122(3)
237.7(1.2)	4.69235(9)	5.69518(14)	4.94839(11)	90.125(3)
257.6(1.1)	4.69303(9)	5.69551(14)	4.94916(11)	90.122(4)
277.4(1.1)	4.69375(9)	5.69617(14)	4.94980(11)	90.122(4)
297.2(1.0)	4.69434(9)	5.69659(13)	4.95052(11)	90.118(4)
316.9(1.0)	4.69505(9)	5.69737(14)	4.95115(11)	90.126(4)
337(4)	4.69572(9)	5.69812(14)	4.95189(12)	90.128(4)
370(4)	4.69694(10)	5.69892(14)	4.95300(12)	90.136(4)
404(5)	4.69800(11)	5.69999(14)	4.95430(14)	90.143(4)
438(5)	4.69925(10)	5.70124(15)	4.95576(15)	90.159(3)
470(8)	4.70048(11)	5.70237(15)	4.95697(15)	90.172(3)

Rietveld Refinements

T(K)	R_p	R_{wp}	R_{exp}	χ^2	R_{Bragg}
2-470	5.74-8.69	8.12-11.9	2.01-4.08	8.04-18.1	17.8-21.5
